# Cerebellar grey matter modifications in lower limb amputees not using prosthesis

**DOI:** 10.1038/s41598-017-18772-2

**Published:** 2018-01-10

**Authors:** Antonella Di Vita, Maddalena Boccia, Liana Palermo, Federico Nemmi, Marco Traballesi, Stefano Brunelli, Roberto De Giorgi, Gaspare Galati, Cecilia Guariglia

**Affiliations:** 1 PhD program in Behavioural Neuroscience, “Sapienza” University of Rome, Rome, Italy; 2 I.R.C.C.S. Santa Lucia Foundation, Rome, Italy; 3 Department of Psychology, “Sapienza” University of Rome, Rome, Italy; 4 Department of Medical and Surgical Sciences, Magna Graecia University, Catanzaro, Italy; 5 Klingberg Lab, Neuroscience Department, Karolinska Institute, Stockholm, Sweden

## Abstract

Plastic brain changes following peripheral deafferentation, in particular those following limb amputations, are well-documented, with significant reduction of grey matter (GM) in the sensory-motor cerebral areas representing the amputated limb. However, few studies have investigated the role played by the use of a prosthesis in these structural brain modifications. Here we hypothesized that using a functional prosthesis that allows individuals to perform actions may reduce grey matter reduction. We investigated the brain structural reorganization following lower limb amputation by using a Voxel Based Morphometry (VBM) analysis of structural magnetic resonance imaging (MRI) in 8 right-handed individuals with lower limb amputation (LLA) fitted with prostheses (LLAwp), compared to 6 LLA who had never used a prosthesis (LLAnp). 14 age-matched healthy controls were also enrolled (HC). We did not find any significant effect when comparing LLAwp and HC. However we found a decreased GM volume in the bilateral cerebellum in LLAnp compared with HC. These results suggest that prosthesis use prevents GM decrease in the cerebellum after lower limb amputation.

## Introduction

One of the most prominent consequences of limb amputation in humans concerns the functional reorganization of the primary somatosensory cortex, with an expansion of adjacent cortical representational areas^1,2^. However, a few studies have also shown structural reorganization in other regions (see Table 1 for a review). Specifically, Draganski and colleagues^3^ found a decrease of thalamic grey matter following limb amputation, whereas Preißler and colleagues^4^ showed a significant decrease of grey matter volume in the left primary motor cortex and in the right dorsolateral prefrontal cortex of patients with right upper limb amputation. Additionally, Xie and colleagues^5^ found an altered interemispheric relationship between the thickness of the postcentral somatosensory areas in eight unilateral lower limb amputees, and Jiang and colleagues^6^ found that lower limb amputees showed decreased cortical thickness in V5/MT + visual areas. Instead, Jiang *et al*.^7^ only found a trend for a reduced cortical thickness in the left premotor area and in visuomotor regions in right lower amputees, and in a small sample of lower limb amputees Hashim, Rowley, Grad, Bock^8^ did not find differences either in cortical thickness or in myelinated thickness in the M1 area representing the lower leg.

**Table 1:** Review of the previous studies on brain structural modifications following limb amputation. Notes. Mth = months; DLPFC = dorsolateral prefrontal cortex; VBM = Voxel Based Morphometry; — not investigated.

Paper	Sample size	Amputation localization (upper/lower limb)	Time from amputation (range)	MRI method/brain area	Results: Amputee participants (AP) vs Control participants (CP)	Grey matter change?	Correlation between grey matter volume and prosthesis use?
Draganski *et al*.^3^	28	16 left lower; 8 right lower; 4 left upper, 2 right upper, 1 left lower and upper	9–444 mth	VBM	*Decreased* thalamic grey matter	Yes	—
Preissler *et al*.^4^	21#	21 right upper	1–600 mth	Cortical reconstruction	*Increased* volume in left temporal pole, left dorsolateral prefrontal cortex (DLPFC), left fusiform cortex, right middle temporal cortex, and the right superior parietal cortex. *Decreased* volume in the left primary motor cortex and in the right DLPFC	Yes	—
Preissler *et al*.^10^	21#	21 right upper	1–600 mth	Cortical reconstruction	—	—	Yes: *negative* with volume in left intraparietal and superior parietal sulci, right middle temporal gyrus, left cuneus, *positive* with volume in right lingual gyrus
Xie *et al*.^5^	8	8 lower limb (no details on side)	4.8–180 mth	Interhemispheric relationships of thicknesses in postcentral somatosensory cortex (PCS) and lateral occipital visual cortex	PCS thicknesses in the left and right hemispheres positively related in CP, but not in AP. The range of the PCS interhemispheric thickness differences in AP was larger than CP.	Yes	—
Hashim *et al*.^8^	4	2 lower left, 2 lower right	444–780 mth	Myelinated thickness and cortical thickness in the area representing the lower leg in M1	No statistically significant in the myelinated thickness and in cortical thickness	No	—
Jiang *et al*.^7^	17	17 lower right	7–336 mth	Cortical thickness and diffusion tractography	Tendency in *decreased* volume in left premotor area and in visual-to-motor regions	Trend	—
Jiang *et al*.^6^	48	26 right lower; 22 left lower	1–336 mth	Cortical thickness in specific visual areas	*Decreased* thickness in V5/MT+	Yes	—
Current series	14	5 right lower; 9 left lower	1.8–250.4 mth	VBM	*Decreased* cerebellar volume in AP without prosthesis.No differences between CP and AP with prosthesis.	Yes, only in AP without prosthesis	Yes, *positive* with volume in left cerebellum

^#^The same sample of participants has been enrolled in Preissler *et al*.^4^ and Preissler *et al*.^10^.

Differences across studies could be due to methodological factors such as sample size, site of amputation (lower vs. upper limb), time since amputation, MRI method (use of region of interest or surface-based methods instead of a volumetric approach; see Table 1 for details on these variables in different studies). In general, these studies suggest that limb amputation yields some structural modifications, even if some inconsistencies in results are evident and only very few studies attempted to systematically analyze the role of specific factors on the variability described in the structural reorganization.

A factor that has been suggested to be pivotal in affecting brain changes after limb amputation is the use of a prosthesis. Lotze, Flor, Grodd, Larbig and Birbaumer^9^ found that enhanced use of a myoelectric prosthesis in upper extremity amputees was associated with reduced functional cortical reorganization. However, only one study has investigated the effect of using a prosthesis on structural brain reorganization. Specifically, in a sample of patients with right upper limb amputation Preißler *et al*.^10^ found negative correlations between the amount of prosthesis use and grey matter volume in the left intraparietal sulcus, the left superior parietal lobe, the right middle temporal gyrus, and the right cuneus, and positive correlations with the cortical volume in the right lingual gyrus. These authors advanced some interesting hypotheses to explain these striking negative correlations. In their study, the prosthesis did not include a flexible wrist and did not provide somatosensory feedback, resulting in a loss of fine tuning during grasping and in the possibility that, the more the patient used the prosthesis, the more he learned that proprioceptive and visual information did not converge during grasping. This in turn could result in grey matter reduction in the posterior part of the parietal lobe involved in these grasping skills. The authors also suggested that, with increasing prosthesis use, amputees could rely more often on top-down instead of bottom-up, stimulus-driven, control of attention, resulting in a reduction in the volume of structures involved in bottom-up control such as the cuneus.

Studies on the effect of prosthesis use on structural brain reorganization in lower limb amputees are missing. However, considering that brain circuits devoted to the motor control of the upper and lower limbs are different, the brain reorganization in this population could be different from that of upper limb amputees as studied by Preißler and colleagues^10^.

Here we aimed to test whether lower limb amputation yields a quantitative variation of grey matter volume as a function of prosthesis use. To this aim we use a Voxel Based Morphometry (VBM) analysis of structural magnetic resonance imaging (MRI). Based on the results of the previous studies, we expected to find specific effects of prosthesis use in the brain networks involved in motor execution and body representation^11^, namely reduced effects of gray matter alteration in individuals who use the prosthesis relative to those who do not use a prosthesis.

## Results

### Between-group comparison

**Figure 1:**
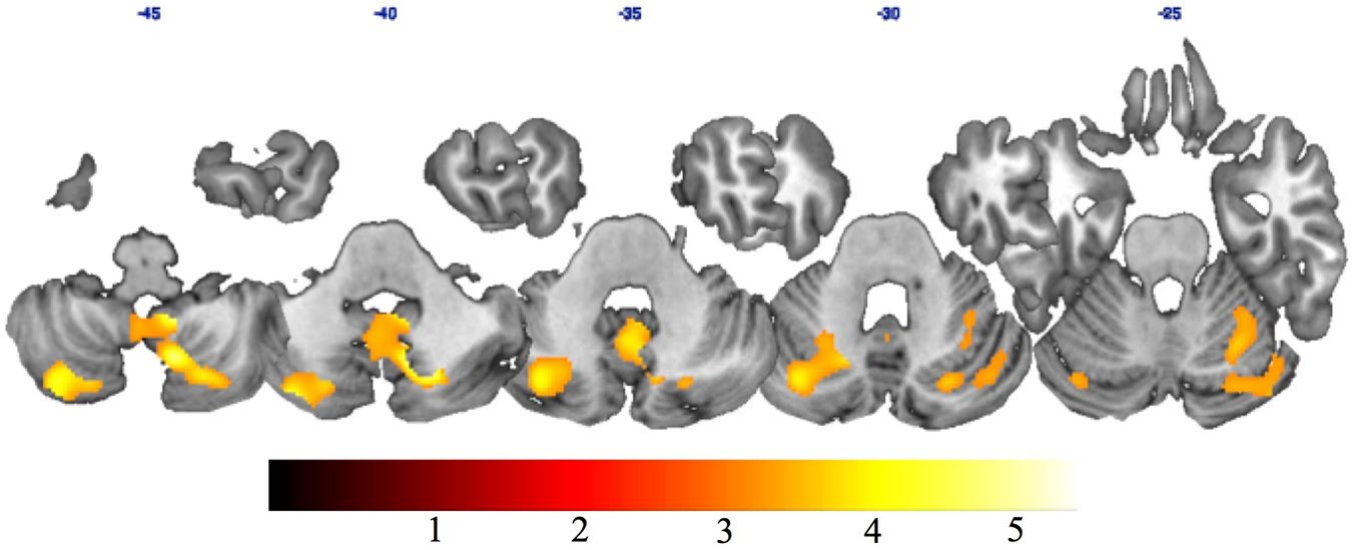
The red-to-yellow patches show, on axial slices, the t statistic of the comparisons between grey matter volume of HC and LLAnp for p < 0.05, corrected for multiple comparisons at the cluster level using false discovery rate and peak p < 0.001 uncorrected.

First, we compared smoothed GM images of the right and the left LLA. No significant suprathreshold voxel was detected between individuals with right and left lower limb amputation (LLA). Thus, we mirrored the MRI scans of the patients with an amputation on the right side in the sagittal plane in order to normalize all patients to one side (see Draganski *et al*.^3^ for a similar methodology). Then, we compared smoothed GM images of LLA and healthy controls (HC): no suprathreshold voxel was detected when we directly compared LLA with HC. Finally, we subdivided the LLA group according to use of a prosthesis and compared LLA who never used a prosthesis (LLAnp), patients fitted with a prosthesis (LLAwp) and HC: we found a decreased GM volume in the bilateral cerebellum in LLAnp compared with HC, mainly located in the VIII lobe extending to the IX lobe in the right cerebellum, and in the Crus II extending to Crus I and VIIb lobe in the left cerebellum (Fig. 1; Table 2). No differences were detected between HC and LLAwp. A trend of decreased GM volume in the left cerebellum (MNI: −34, −73, −35) in LLAnp with respect to LLAwp was also observed, even if this difference did not survive multiple comparisons correction at the cluster level.

**Table 2:** The table lists the regions showing higher gray matter volume in controls than LLA without prosthesis, the hemisphere, the T-score (p FDR-corrected < 0.05), the region volume (voxels), the peak p value and the MNI coordinates.

Region	Hemisphere	cluster p (FDR-corr)	T	Volume (k)	peak p (unc)	x	y	z
*Control* > *LLA without prosthesis*								
*Cerebellum VIII*	*R*	*0.000*	*5.80*	*2157.00*	*0.000*	11	*−*65	*−*44
Cerebellum IX	R		5.04		0.000	11	−50	−45
Cerebellum VIII	R		4.90		0.000	20	−71	−41
*Cerebellum Crus II*	*L*	*0.001*	*4.59*	*1527.00*		*−*35	*−*77	*−*44
Cerebellum CrusI	L		4.45			−33	−72	−33
Cerebellum VIIb	L		4.11			−24	−74	−44

### Correlations analyses

**Figure 2:**
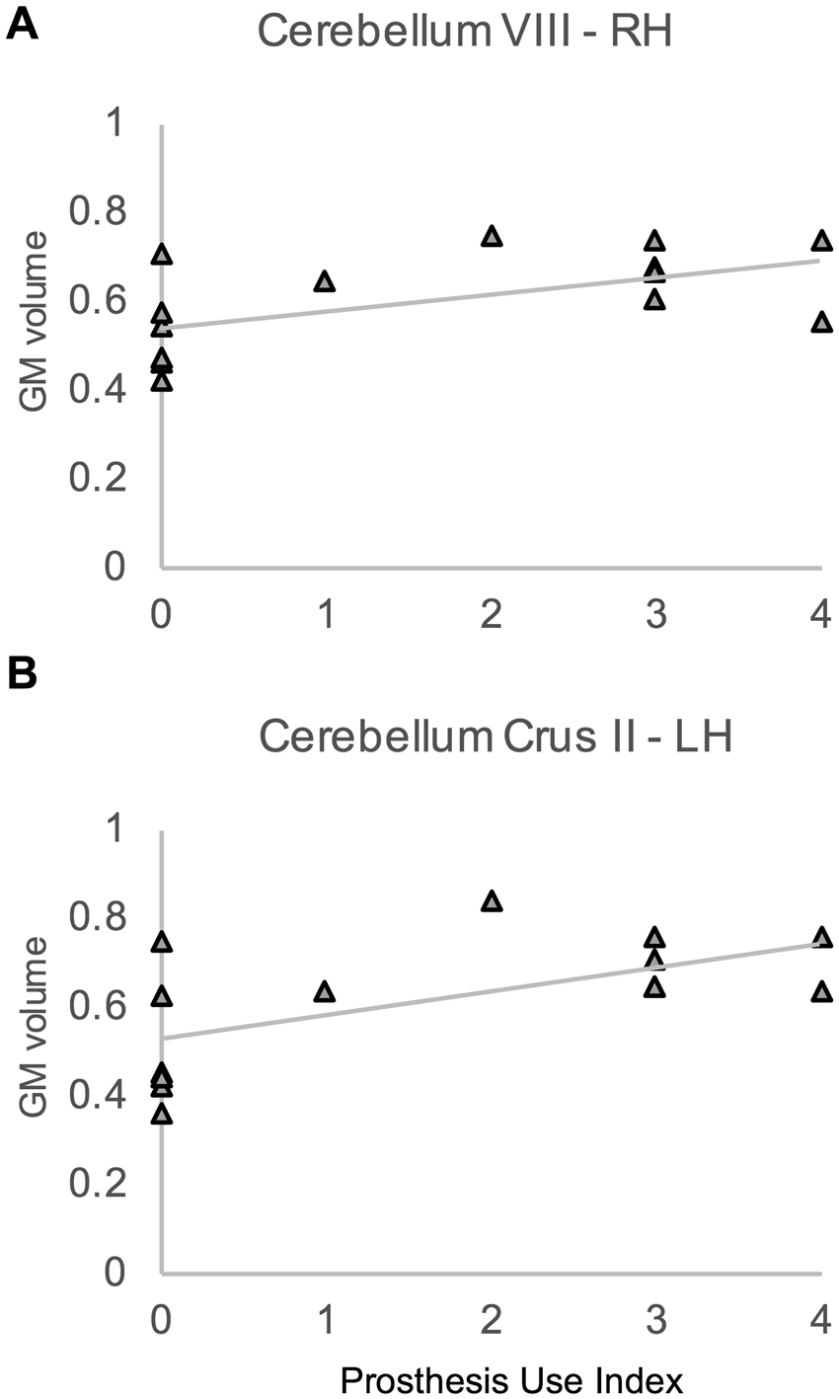
Scatter plots depict the correlation between prosthesis index and GM volume in cerebellar lobules VIII (**A**) and CrusII (**B**). Each triangle represents a participant of the LLA group.

To further assess the effect of prosthesis use on GM volume of LLA, we performed correlation analyses between GM volume and an index of prosthesis use calculated with a procedure inspired by Preißler *et al*. (see the method section below for further detail). The average GM volume in the cerebellar clusters identified in group comparison (see Table 2) significantly correlated with the daily amount of prosthesis use expressed by the prosthesis index. Both for the cluster in the right cerebellum lobe VIII (τ = 0.0407; one tail p = 0.031) and for the one in the left cerebellum lobe Crus II (τ = 0.432; one tail p = 0.024), the correlation was positive, namely higher GM volume was associated with higher prosthesis use (see Fig. 2). We additionally performed a partial correlation analysis to regress out possible spurious effect due to the time since amputation. The results of this analysis were consistent with the former confirming that higher prosthesis use was associated with higher cerebellar volume in Crus II and lobe VIII (supplementary Table 1).

## Discussion

Here we investigated the neural modifications following lower limb amputation and whether the use of a functional prosthesis affects these modifications.

The VBM results did not show any significant difference when comparing HC and LLA as a whole. Instead, a decrease in GM volume was observed in LLAnp as compared to HC in the right cerebellar lobes IX and VIII as well as left cerebellar lobes Crus I, Crus II, and VIIb. The results are in line with those by Lotze *et al*.^9^, who showed a reduced cortico-functional reorganization in prosthesis users.

The significant decrease of GM volume detected only in LLAnp suggests that the structural modifications following lower limb amputations are related to the lack of prosthesis use. Indeed, the GM volume of LLAp did not differ from that of HC, suggesting that the use of a functional prosthesis may prevent the GM decrease in the area representing the now amputated lower limb and its movements.

Overall, the present results suggest that the deafferentation (without the prosthesis use) induces a “negative” alteration, while the stimulation of afferent input induces a functional expansion of adjacent cortical representational areas (see for example Draganski *et al*.^12^), that is a “positive” alteration. The mechanisms subserving these modifications are not yet completely understood^3,7^ and deserve further investigations.

For the first time, our work demonstrates in a group study the involvement of cerebellar alteration in amputees, linked to the prosthesis use, which is likely due to the different alterations of somatosensory inputs between those who do not use the prosthesis and those who do use it. Indeed, according to the literature review described above, only the Gaser, Nenadic, Weiss, Miltner & Sauer’s^13^ single case study documented a continuous grey matter loss in the first 21 weeks after amputation also in the cerebellum. Accordingly, Mizelle, Oparah &Wheaton^14^ highlighted a downregulation of cerebellar activity when somatosensory feedback was altered without an adaptation period in a study investigating the role of visual and somatosensory feedback in skilled movements (i.e. participants used tweezers to move a cube through the quadrant of a board, following a given sequence). To reduce the reliability of somatosensory information, they induced a transient ischemic deafferentation of the distal right arm during a tool-use motor task and, to reduce the reliability of visual information, they modified the clarity with which participants saw. In the condition with unreliable somato-sensation, a reduction in cerebellar activation and an expansion of bilateral cortical sensorimotor and temporo-occipital junction activations were found.

At variance with some of the studies mentioned above (see also Table 1), the present VBM’s results did not show any modification in cortical areas but only in subcortical ones. However, exclusive subcortical modifications have been also found by Draganski *et al*.,^3^ who suggested that the functional reorganization that occurs at the cortical level^15–17^ may balance the neuronal atrophy.

In particular, our study did not find any involvement of the primary motor and/or somatosensory cortex. This result may be due to the fact that lower limbs are less represented in the somatomotor cortex^7^ with respect to the upper ones.

Interestingly, it must be considered that the cerebellum is a critical region for sensorimotor functions and motor behavior. Although the cerebellar areas identified in the present study are globally located in what is considered the posterior cerebellum, classically involved in higher-level processes and therefore labelled as the “cognitive cerebellum”^18^, recent evidence supports the idea that these areas (i.e. VIIb, VIIIa/b, X, and Crus I) are involved in somatomotor functions, especially when visual and somatosensory information are compromised^14^. Additionally, multiple somatotopic maps have been identified within the cerebellum (see review in Manni and Petrosini^19^; Bernard and Seidler^20^; Grodd, Hulsmann, Lotze, Wildgruber and Erb.^21^; Buckner, Krienen, Castellanos, Diaz and Yeo^22^). The results of these studies revealed two body representations in the cerebellum, one in the anterior lobe (lobules I–V) and one in the posterior lobe, in particular in lobules VIII and IX^20–22^. Interestingly, the localization of the somatomotor representation of the foot by Buckner and colleagues^22^ roughly corresponds to the grey matter reduction we found in LLAnp. This cerebellar region^22^, and Crus II as well, have been shown to have connections with associative cortical areas devoted to body representation in the posterior parietal cortex^23,24^. Among these areas, the supramarginal gyrus has been found to be selectively associated with a body representation not involved in action (non-action body representation, NA^11^), that is with those mental body representations which are not evoked during action but, for example, process the spatial/metric aspects of the body. The association between amputation and modification of NA is further supported by neuropsychological data showing that lower limb amputation may yield a deficit in visuo-spatial representation of body parts^25^.

Cerebellar lobules single out by the analysis reported here (e.g. lobule VIII) are linked to cortical association areas^22^, which have functional laterality. Considered that, as reported in the method section, we mirrored the MRI scans of the patients with an amputation on the right side in order to normalize all patients to one side, the present data about lateralization should be considered with caution.

Interestingly, posterior cerebellum has been found to be implicated in learning the use of a new tool. It has been proposed that the activity of lobule VIII, contributes to the implementation of internal models^26–28^, that is the mechanism that allows for storing objects and body properties, needed to acquire skillfully manipulation of objects^27^. A previous study in right upper limb amputees^10^, by using cortical reconstruction and volume segmentation, found a negative correlation between the volume in the left posterior parietal cortex and the amount of prosthesis use. In that study, less prosthesis use was associated with phantom limb pain. The difference with the present results may be due to at least two important factors other than the simple difference in samples (i.e. upper limb vs. lower limb amputees), that is (1) the use of a different methodological approach (i.e. cortical reconstruction vs. VBM); (2) the fact that the use of a prosthesis in our sample was not linked to the absence/presence of phantom limb pain.

Finally, to the best of our knowledge, this is the first time that the effect of the prosthesis on GM volume has been assessed in lower limb amputees by taking into account the effective amount of prosthesis use. We found a positive correlation between GM volume of Crus II and VII cerebellar lobules and daily use of the prosthesis (hours/day).

We acknowledge that a main limitation of this study was its relatively small sample size. We focused our investigation on an homogeneous sample, namely lower limb amputees, in order to control for spurious effects related to the amputation site (e.g. lower vs. upper limb amputation), but this choice unavoidably affected the sample size.

In summary, this study provides the first evidence for cerebellar modifications in amputees who are not using a prosthesis. The results suggest that prosthesis use prevents GM decrease in cerebellum and yields cerebellar modification as a function of the total amount of prosthesis use. These preliminary findings may also provide relevant implications for improving the clinical management of these patients, but further investigations on larger samples are necessary before drawing definitive conclusions.

## Method

### Participants

Participants included 14 right-handed individuals with lower limb amputation (LLA; mean age: 45.50 and SD: 17.90) and 14 healthy age-matched controls (HC; mean age: 39.79 and SD: 14.58; t (26) = −0.35; p = 0.36). All participants were male and none of them had any history of neurological or psychiatric disorders.

All participants showed normal reasoning skills assessed by means of an abstract reasoning test^29,30^, commonly used to exclude the presence of general cognitive deficits.

In the LLA group the average time since amputation was 1631 days (SD 2197; minimum 53 days; maximum 7511 days). The amputation, due in most cases to trauma (n = 9), was located on the left side in 9 and on the right side in 5 cases. Phantom limb phenomena were present in all 14 LLA. Eight out of 14 LLA had been fitted with prostheses (LLAwp) while six LLA had never used a prosthesis (LLAnp). LLAwp and LLAnp did not differ in age (t (12) = −0.80; p = 0.43) or time since amputation (t (12) = −0.10; p = 0.92; LLAnp’s minimum = 53 days; LLAnp’s maximum = 7511 days; LLAwp’s minimum = 102 days; LLAwp’s maximum = 5211 days). All the LLAwp used the same kind of prosthesis (i.e. Endoskeletal modular prosthesis with suction suspension system). The prosthesis use was not associated with the presence of phantom limb pain (Chi-square = 0.22; p = 0.64). For more details about demographics and clinical data on the LLA group see Table 3.

**Table 3:** Demographics.

No	Age	Handedness	Amputation		Time since amputation (days)	Cause	Prothesis Use (hours/days)	
			Left	Right				
1	48	Right		x	1933	Traumatic	no	0
2	53	Right		x	55	Vascular	no	0
3	79	Right		x	742	Vascular	yes	12
4	57	Right		x	151	Vascular	no	0
5	51	Right		x	2202	Traumatic	yes	20
6	21	Right	x		184	Traumatic	yes	4
7	65	Right	x		102	Tumor	yes	3
8	47	Right	x		1770	Traumatic	yes	12
9	27	Right	x		484	Traumatic	yes	24
10	33	Right	x		7511	Traumatic	no	0
11	18	Right	x		1874	Traumatic	yes	16
12	29	Right	x		5211	Traumatic	yes	9
13	63	Right	x		556	Vascular	no	0
14	46	Right	x		53	Traumatic	no	0

All participants gave their written informed consent to participate in the study that was designed in accordance with the principles of the Declaration of Helsinki and approved by the ethical committee of IRCCS Fondazione Santa Lucia of Rome.

### Image Acquisition

A Siemens Allegra scanner (Siemens Medical Systems, Erlangen, Germany), operating at 3 T was used to acquire magnetic resonance images. Head movements were minimized with mild restraint and cushioning. We acquired a three-dimensional high-resolution T1-weighted structural image for each subject (Siemens MPRAGE, 176 slices, in-plane resolution 0.5 × 0.5 mm^2^, slice thickness 1 mm, TR 2 s, TE 4.38 ms, flip angle 8 deg).

### Voxel based morphometry analysis (VBM)

As we would not restrict our investigation to specific brain structures or regions, neither to local cortical surface, we used a Voxel Based Morphometry to investigate our hypothesis. We performed a VBM analysis on participants’ T1-weighted structural images, using the VBM8 Toolbox, implemented in SPM8. The T1 anatomical images were manually checked for scanner artifacts and gross anatomical abnormalities. The images were then normalized using high-dimensional DARTEL normalization, segmented into grey matter (GM), white matter (WM), and cerebrospinal fluid (CSF), and smoothed (FWHM 8 mm). A two-sample t-test was used to compare the smoothed GM images of (1) the right and the left LLA, and (2) those of LLA and HC individuals. We also performed (3) a one-way ANOVA to compare smoothed GM images of HC, LLAwp and LLAnp. The resulting statistical parametrical maps were thresholded at p < 0.05, corrected for multiple comparisons at the cluster level using false discovery rate^31^, after forming clusters of adjacent voxels surviving a threshold of p < 0.001 uncorrected.

To assess the effect of the amount of the prosthesis use, we performed a non parametric correlation (Kendall’s tau; τ) between the average GM volume in the clusters raised from the previous comparison and a prosthesis use index calculated with a procedure inspired by Preißler *et al*.^10^. In particular, a five point categorical scale was used to classify daily amount of prosthesis use with 0 = never, 1 = 1–3 hours, 2 = several hours but not continuously, 3 = continuously for either the whole morning or the whole afternoon, and 4 = from morning to night.

## Electronic supplementary material

Supplementary Table S1
