# Supplementary material for: Cerebellar grey matter modifications in lower limb amputees not using prosthesis
Source: Sci Rep. 2018 Jan 10;8:370. doi: 10.1038/s41598-017-18772-2 (PMC5762812; doi:10.1038/s41598-017-18772-2)
Supplement: Supplementary file 1 — Supplementary Table S1 [file 41598_2017_18772_MOESM1_ESM.pdf]

Supplementary Information

Cerebellar grey matter modifications in lower limb amputees not using prosthesis

Antonella Di Vita, Maddalena Boccia, Liana Palermo, Federico Nemmi, Marco Traballesi, Stefano Brunelli, Roberto De Giorgi , Gaspare Galati, Cecilia Guariglia

		IPU	Cerebellum VIII	Cerebellum Crus II
IPU	*r*	1.000	***.561***	***.610***
	p		***.023***	***.013***
Cerebellum VIII	*r*	***.561***	1.000	.959
	p	***.023***		.000
Cerebellum Crus II	*r*	***.610***	.959	1.000
	p	***.013***	.000	

Supplementary Table S1 – Partial correlations between IPU and GM volume of Cerebellum VIII and Cerebellum Crus II after regressing out the effect of the time since amputation. IPU= Index of Prosthesis Use
